# Objective Standards of Medical Judgment: A Myth of (Texas) Abortion Law

**DOI:** 10.1017/jme.2025.10208

**Published:** 2026

**Authors:** Abraham Graber, Mack Peterson, Ethan Detrick

**Affiliations:** 1 Biomedical Education and Anatomy, College of Medicine, The Ohio State University Wexner Medical Center, Columbus, United States; 2 The Ohio State University, United States

**Keywords:** Abortion, *Dobbs v. Jackson*, Human Life Protection Act, Objective Standards, Subjective Standards

## Abstract

Post-*Dobbs v. Jackson*, abortion regulation is left entirely to the states. Laws that restrict access to abortion generally allow for exceptions when determined necessary for the life or safety of the pregnant patient. Some states, e.g., Ohio, use a “subjective” legal standard when determining whether an abortion is medically necessary. Other states, e.g., Texas, rely on an “objective” legal standard, whereby the necessity of an abortion is not determined by any particular physician’s judgments, but rather by the judgment of a hypothetical “reasonable physician.” Though objective legal standards are widespread in American jurisprudence, they are a poor fit for clinical judgments about the medical necessity of abortion. On the contemporary model of clinical decision-making, medical judgment is irremediably subjective. In addition to being responsive to patient values and medical evidence, medical judgment is, and should be, informed by physician values. Because Texas abortion regulations rely on an objective standard of judgment that fails to correspond to a medically meaningful category, they fail to provide adequate guidance to physicians regarding the circumstances under which abortion is legally protected.

## Introduction

*Dobbs v. Jackson* overturned the long-standing constitutional right to abortion established by *Roe v. Wade*, leaving states solely responsible for the regulation of abortion access.^1^ As of September 2025, nineteen states have introduced new restrictions on abortion access.^2^

Abortions can protect the health, and save the life, of pregnant patients. Laws restricting abortion access thus generally include exceptions for abortions deemed medically necessary.^3^ Relatedly, efforts to protect abortion access often follow the example set by *Roe v. Wade* and allow restrictions on abortion access after fetal viability. Such laws nonetheless often preserve a right to post-viability abortion when one is deemed medically necessary.^4^

Recent reporting has, however, put a spotlight on legal clauses that aim to protect abortion access in those cases where an abortion may be medically necessary. For example, work by ProPublica has highlighted maternal deaths in Texas that could have been prevented by the provision of an abortion.^5^ Some Texas physicians have argued that these deaths were the result of ambiguity in Texas law regarding when an abortion can legally be performed.^6^ Legal scholars have, relatedly, highlighted the complexities of obstetric care and contended that this complexity makes the relevant legal ambiguity intractable.^7^ On the other side of the debate, supporters of Texas’ abortion regulations have countered that there is no such ambiguity.^8^ The Texas Supreme Court concurred with this latter view, ruling that Texas regulations are not ambiguous regarding the conditions under which an abortion can legally be provided.^9^

Contra the Texas Supreme Court, we argue that Texas abortion regulations fail to provide adequate guidance to physicians who encounter patients with pregnancy complications. In its decision in *State v. Zurawski*, the Texas Supreme Court’s conclusion that Texas abortion regulations are not ambiguous rested largely on the Human Life Protection Act’s use of an objective legal standard regarding what constitutes a threat to a patient’s life or health.^10^ In what follows we will argue that Texas law and the Texas Supreme Court’s decision in *State v. Zurawski* are grounded in a misunderstanding of the nature of medical practice. In particular, we will argue that the objective legal standard of clinical judgment found in the Human Life Protection Act, and affirmed by the Texas Supreme Court, fails to correspond to a meaningful medical category.

Though our central analysis focuses on Texas abortion regulations, the implications for abortion regulation are much broader. Texas abortion regulations serve as a case study that illustrate a mismatch between the contemporary understanding of medical practice dominant in medicine and in medical ethics and the reliance on objective legal standards found in abortion regulation. This mismatch entails that there is substantial and ineliminable ambiguity inherent in attempts to rely on objective standards of medical judgment for determining when an abortion is necessary to protect the health or life of a patient.

## Objective vs. Subjective Standards

In America, the distinction between objective and subjective standards plays a central role in civil, criminal, constitutional, contractual, and commercial law.^11^ A “subjective standard depends on the state of mind of the actor,”^12^ or, in the context of Texas abortion regulation: “A subjective standard … examines a doctor’s intent instead of medical facts.”^13^ By contrast, “an objective standard focuses on the actor’s behavior; a given act is permissible or not, regardless of what the actor had in mind.”^14^

Ohio and Texas abortion regulations helpfully illustrate the distinction. In Ohio, abortion access is protected until the point of fetal viability. After viability, abortion access can be restricted; however, “in no case may such an abortion be prohibited if *in the professional judgment of the pregnant patient’s treating physician* it is necessary to protect the pregnant patient’s life or health.”^15^ The Ohio Constitution deploys a subjective standard where the physician’s state of mind is key for whether a post-viability abortion is legally protected.

By contrast, in Texas, access to abortion remains legal when:

[I]n the exercise of *reasonable medical judgment*, the pregnant female on whom the abortion is performed, induced, or attempted has a life-threatening physical condition aggravated by, caused by, or arising from a pregnancy that places the female at risk of death or poses a serious risk of substantial impairment of a major bodily function unless the abortion is performed or induced.^16^ (emphasis added).

Texas’s Human Life Protection Act employs an objective standard. What matters is not the state of mind of the treating physician, but rather “reasonable medical judgment.” “Reasonable medical judgment” is defined as “a medical judgment made by a reasonably prudent physician, knowledgeable about a case and the treatment possibilities for the medical conditions involved.”^17^ In turn, “a reasonably prudent physician” is generally understood as a physician with the “degree of skill and learning commonly possessed by members of the medical profession.”^18^ Thus, in Texas, the legality of an abortion performed to protect a patient from death or substantial impairment does not depend on the professional judgment of the treating physician. Rather, it depends on the judgment that a hypothetical “reasonably prudent physician” would make.

## The Contemporary Model of Clinical Decision-Making

Central to *State v. Zurawski* was a disagreement over which kind of standard — objective or subjective — it was appropriate to use in making the determination that an abortion is medically necessary. In order to answer this question, one must go beyond legal standards and ask: How does modern medicine understand the nature of appropriate medical judgment? The model of medical judgment at the heart of contemporary medicine views clinical judgments as responsive to at least three distinct variables.

First, appropriate clinical judgment is constrained by scientific evidence.^19^ In some cases, there may be widespread agreement about the available evidence among physicians with the “degree of skill and learning commonly possessed by members of the medical profession.” As such, this component of clinical decision-making can sometimes be assessed by an objective standard of “reasonable medical judgment.”

This first aspect of appropriate clinical judgment helps to make sense of reliance on objective standards of clinical judgments in malpractice cases.^20^ There is a wealth of scientific evidence relevant to the practice of medicine. This evidence highlights, e.g., appropriate sterilization techniques for surgical instruments, the potentially fatal results of high doses of opioid medications, and the inability of ivermectin to treat COVID-19. The evidence base on which medicine is built allows for many medical decisions to be judged via an objective legal standard.

It should be noted that, while there are cases in which the available evidence demands agreement amongst reasonably prudent physicians, it is unclear how often medical decisions regarding pregnancy complications fall under this category. With regard to pregnancy complications, physicians are often working under conditions of substantial diagnostic and prognostic uncertainty such that there is unlikely to be agreement amongst reasonably prudent physicians.^21^ Under such conditions, medical judgments plausibly cannot be evaluated via reference to an objective standard. The evidential ambiguity common in clinical contexts presents an important concern regarding the appropriateness of assessing medical judgments via objective standards.

In what follows we will assume that the component of medical judgment informed by the available scientific evidence can be assessed via an objective standard. Doing so best facilitates the exploration of a distinct source of ambiguity that may undermine the use of objective standards in abortion regulation. That said, the assumption that medical and scientific facts are unproblematically assessed via an objective standard should be understood as the bracketing of a distinct concern for the purposes of expositional clarity, and not as an expression of the authors’ considered views on the matter.

Second, respect for patient autonomy requires that medical judgment be informed by the values and preferences of the patient.^22^ Thus, even given identical diagnoses and prognoses, a physician’s medical judgment regarding appropriate treatment may vary substantially from one patient to the next. This aspect of clinical decision-making can also be assessed against an objective standard. An objective standard assesses physician behavior by reference to something other than “the state of mind of the actor, [i.e., physician].”^23^ The values in question here are the values of the patient, not the physician. Thus, whether a physician’s clinical judgment is appropriately responsive to a patient’s values can be assessed via an objective standard.

The same cannot, however, be said regarding the final component of the contemporary understanding of appropriate clinical judgment. On our current understanding of medicine, medical judgment must also respect a physician’s values. Though the literature supporting the centrality of physician values to appropriate medical judgment is too voluminous to sketch here, it can nonetheless be helpful to briefly summarize one line of reasoning that has led to this consensus.

The contemporary view that physician values should play a central role in medical judgment arises, in part, from the shift away from the parentalism — whereby the physician is understood to know best and the patient’s role is merely to follow physician directives — characteristic of medical practice prior to the 1970s and 1980s. Contemporary medicine’s rejection of parentalism is realized by an emphasis on respect for patient autonomy, sometimes described as “first amongst equals” regarding the considerations relevant to medical decision-making.^24^ Respect for patient autonomy is, however, only one of several considerations relevant to medical judgment. In addition to autonomy, physicians must also prioritize non-maleficence (i.e., avoiding causing harm), beneficence (i.e., benefiting the patient), and justice.^25^

In our contemporary, pluralistic society, physicians cannot expect unanimity regarding what counts as a benefit or as a harm.^26^ For example, while disabilities are widely viewed as a harm, members of the Disability Pride movement strongly reject this characterization and sometimes try to increase the probability that their child will be born disabled.^27^ Relatedly, rooted in their view that disease is an illusion, Christian Scientists are likely to disagree with physicians regarding what counts as beneficent or non-maleficent.^28^ Examples abound.

In the face of pluralism regarding value, how should physicians assess whether an intervention is beneficent or non-maleficent? Using a patient’s value system effectively collapses beneficence, non-maleficence, and respect for autonomy, leaving a patient’s values and preferences as the only compass regarding appropriate medical judgment. Furthermore, the pluralistic nature of our society entails that, outside of a patient’s value system, there is no single, unified value system to which the physician can refer:^29^ In the words of the President’s Commission, “[W]ell-being is not a concrete concept that has a single definition.”^30^ Furthermore, both the view that the appropriate boundaries of medicine are set by the dyadic physician-patient relationship^31^ and the contemporary emphasis on patient autonomy preclude solutions that navigate the challenge by introducing values external to either the physician or patient. Thus, for example, democratic solutions to the challenge of value pluralism are not viewed as viable candidates in the context of medical decision-making. Instead, contemporary medicine maintains beneficence and non-maleficence as guides to medical judgment and prevents them from collapsing into respect for patient autonomy by integrating physician values into medical judgment.

Maintaining a distinction between autonomy on one hand, and beneficence on the other, is not merely of theoretical importance. The physician’s obligation to respect autonomy is understood to be a negative obligation, i.e., an obligation of non-interference.^32^ Respect for patient autonomy thus requires that physicians not do anything to a patient’s body without either the patient’s consent or the consent of an appropriate proxy; it does not, however, require that physicians do what a patient requests.^33^ By contrast, beneficence is understood to be a positive obligation; physicians are required to take action to help their patients.

If judgments about what constitutes a patient’s well-being are left entirely up to the patient’s values, autonomy and beneficence effectively collapse, as “beneficence” becomes nothing more than another avenue for expression of a patient’s values. This is especially problematic because beneficence is a positive obligation, requiring that physicians act so as to improve a patient’s well-being. Thus, failure to include physician values as a key component in medical judgment effectively leaves physicians at the whim of patient values, thereby not only ignoring the physician’s own values, but also their medical expertise regarding what does, and does not, contribute to patient health.

The view that physician values related to well-being are central to medical judgment is, by now, ubiquitous. For example, the President’s Commission (1982) notes that:

Health care professionals often reflect their own value preferences when they favor one [treatment] alternative over another*;* many are matters of choice, dictated neither by biomedical principles or data nor by a single, agreed-upon professional standard.^34^

Kaldjian concurs with this understanding of the practice of medicine:

[A] family may prefer that a patient’s life be prolonged by intensive life-supporting means, whereas the physician or nurse believes that continued life support will … only prolong suffering. Such disagreements remind us that the selection of goals is not only based on biomedical realities and available technology, but also on beliefs about matters as fundamental as the value of prolonging life, the acceptability of suffering, the significance of a given outcome probability, and the financial implications of treatment. As a result, goals may need to be explained or negotiated in the hope that a shared understanding between involved parties will emerge.^35^

As Kaldjian highlights, the role of physician values in medical judgment is central to shared decision-making, the most widely accepted model of the physician-patient relationship. In shared decision-making, the goal is for a physician and patient to “build a consensus,”^36^ i.e., to arrive at an understanding that is acceptable to both the physician and the patient. Furthermore, and contrary to what its name suggests, shared decision-making is now viewed not only as relevant to medical decision-making, but rather as central to the full scope of the physician-patient relationship, from identifying and understanding the problem for which the patient seeks care through to the implementation of intervention.^37^ It follows that on the most widely accepted model of the physician-patient relationship, understood as “the pinnacle of patient-centered care,”^38^ physician values are central to appropriate medical judgment.

Finally, the centrality of physician values to medical judgment is perhaps best highlighted by the fact that even authors who advocate for constraints on physician values in medical care nonetheless accept the centrality of physician values in medical judgment. Consider, e.g., Drescher’s argument that removing physician values from medical judgment is an impossibility:

No physician can claim to practice value-free medicine. Undoubtedly, physicians are raised with values, religious or otherwise, that shape their decisions to become professional caretakers. Their training is further influenced by professional values, embodied in the Hippocratic Oath, the Oath of Maimonides, and the AMA’s Principles of Medical Ethics.… Consequently, I think it unreasonable, if not impossible, to ask physicians to practice “value-free” medicine.^39^

This is not, however, to say that physicians are authorized to unilaterally determine what is in a patient’s well-being. Rather: “[A]ll patients competent to do so have a right to accept or reject medical interventions affecting them.”^40^ Furthermore, while some authors have embraced a much more substantial role for physician values in medical judgment (especially in the literature on conscientious objection),^41^ these more expansive views remain contested.^42^ In particular, insofar as there is widespread agreement in medicine and medical ethics that physician values are relevant to medical judgment, this consensus is specific to physician values related to *harms* and *benefits* within the medical context and does not extend to physician views about the rightness and wrongness (or permissibility and impermissibility) of a patient’s choices or way of being.^43^

By way of illustration, consider recent reports that indicate that a woman was denied prenatal care in Tennessee on the grounds that she is unmarried.^44^ This denial of care appears to turn on the physician’s view that having a child out of wedlock is *wrong.* However, such judgments of rightness and wrongness on the part of the physician fall outside of the contemporary consensus regarding which physician values are relevant to medical judgment. On the grounds that providing extraordinary measures would do more *harm* than *good*, this consensus would allow a physician to refuse to provide extraordinary measures to briefly extend the life of a dying cancer patient. The contemporary consensus does *not*, however, allow a physician to similarly refuse to provide extraordinary measures on the grounds that the patient is gay and the physician believes that being gay is morally impermissible. Similarly, the contemporary consensus might allow for the denial of prenatal care on the grounds that the provision of prenatal care would, somehow, harm the patient. (This view of the harms and benefits of prenatal care is, of course, directly at odds with contemporary medical knowledge; here, the objective components of medical judgment work to further constrain the degree to which physician values can shape medical judgment.) Importantly, the contemporary consensus does *not* justify the denial of prenatal care on the grounds that a physician thinks that it is *wrong* for a patient to have a child out of wedlock.

One final note is worth making before proceeding. We have thus far left “medical judgment” undefined. In so doing, we are following the lead of Texas healthcare regulation, which similarly does not provide a definition of “medical judgment.” Were our argument to rely on a specific definition of “medical judgment,” the argument could be accused of missing the mark by assuming a definition of “medical judgment” that is something other than how it is understood in Texas law. Thus, rather than giving a definition of “medical judgment,” we have given an account of how contemporary medicine understands what is involved in physician-patient interactions and how physicians ought, ultimately, to come to their conclusions. On this model, physician values are infused throughout the physician-patient relationship; medicine is value-laden through and through. Regardless of how “medical judgment” is ultimately defined, so long as that definition is recognizable from the perspective of medicine itself, physician values will remain a central component.

## Illustrating the Contemporary Model of Clinical Decision-Making

The role of physician values in medical judgment can be helpfully illustrated by considering two (hypothetical) case studies. Each case study illustrates not just the relevance of the so-called “medical facts” for clinical judgment, but also the centrality of physician values. Consider the first case study.

### Blood Transfusion

An adult patient with decision-making capacity is referred for a lifesaving surgery that is likely to require a blood transfusion. The patient is a devotedly observant Jehovah’s Witness. The patient has decisional capacity and understands the relevant risks. Nonetheless, the patient consents to the procedure *only if* blood products are not provided.

#### The First Objective Layer: Scientific Facts

Scientific facts are central to appropriate clinical decision-making. Relevant facts in this case include the prognosis if no surgery is performed and the prognosis if a surgery is performed without blood products. For the sake of argument, we assume that such facts can be assessed by reference to an objective standard.

#### The Second Objective Layer: Patient Values

It would be both unethical and illegal to provide a blood transfusion against the patients’ wishes. Yet were the patient to have different values and be willing to accept a blood transfusion, moving forward with the use of blood products during the surgery would be medically obligatory. Appropriate clinical judgment thus must reflect the values and preferences of the patient.

#### The Subjective Layer: Physician Values

Two equally skilled surgeons who agree about the probability of success in our *Blood Transfusion* scenario may nonetheless differ in their clinical judgment regarding the appropriateness of performing the procedure. One surgeon may judge that, since the procedure would be lifesaving, the potential benefits outweigh the risks of proceeding without blood products. By contrast, another surgeon may believe that the vocation of medicine requires that physicians prioritize not causing the death of a patient. This physician may judge that it is medically inappropriate to provide the surgery without the use of blood products. Furthermore, because it is rooted in distinct value sets,^45^ this disagreement over the appropriateness of providing the surgery without the use of blood products may be effectively intractable.

Consider a second (hypothetical) case-study:

### Necrotic Hand

An adult patient with decisional capacity presents with widespread gangrenous hand tissue. Amputation is recommended. The patient is a neurosurgeon whose work is their passion. Informed of the risks, they nonetheless request a difficult and risky surgery to save the appendage.

#### The First Objective Layer: The Scientific Facts

If tissue necrosis is too advanced for the hand to be saved, it would be inappropriate to move forward with the surgery. The better the surgical prognosis, the more medically appropriate the surgery. For the sake of argument, we assume that such facts can be assessed via an objective standard.

#### The Second Objective Layer: The Patient’s Values

Given the patient’s informed and capacitated desire for a high-risk procedure to save their hand, it would be permissible for an appropriately skilled physician to move forward with the surgery. This is, however, only the case because of the unique value the patient places on hand function. Were the patient to place less value on hand function, attempting to save the hand would be impermissible.

#### The Subjective Layer: Physician Values

Suppose a surgeon feels that — despite the patient’s values — the risks of attempting to save the hand outweigh the benefits. In this surgeon’s clinical judgment, it would be medically inappropriate to provide the surgery. For a physician who puts comparatively more weight on patient values, attempting to save the hand may be medically appropriate.

Relatedly, physician views regarding the value of life with a disability are relevant to judgments regarding well-being. Thus, a physician who views life with a disability as substantially worse than life without is more likely to be willing to provide the intervention, as they will view a successful surgery as providing comparatively substantial benefit. By contrast, a physician who endorses Disability Pride and thinks that life with a disability is no worse than life without one is less likely to favor attempting the surgery, as they will see fewer benefits resulting from a successful surgery. Once again, physician values constitute an ineliminable aspect of appropriate medical judgment.

## Implications for Objective Standards of Clinical Judgment

Both the *Blood Transfusion* and *Necrotic Hand* scenarios illustrate a foundational aspect of contemporary medical practice: though constrained by both patient values and the available scientific evidence, a physician’s medical judgment nonetheless cannot be separated from physician values. The implications for the use of objective standards of medical judgment are immediate.

Consider either *Blood Transfusion* or *Necrotic Hand.* In these cases, what would be done by “a reasonably prudent physician, knowledgeable about a case and the treatment possibilities for the medical conditions involved?”^46^ The notions of “reasonable medical judgment” and of a “reasonably prudent physician” are supposed to constitute objective legal standards.^47^ Yet on the contemporary understanding of medical practice, physician values are a key component of clinical judgment, leaving clinical judgment centrally and ineliminably subjective. Knowing nothing about the values of the “reasonable prudent physician,” we cannot answer the question: What would the reasonably prudent physician do in *Blood Transfusion* and *Necrotic Hand*?

Importantly, this ambiguity does not arise from the existence of clinical equipoise. When clinical equipoise exists, physicians may disagree about what ought to be done clinically while nonetheless viewing alternative treatment options as medically appropriate. In such cases, disagreement between “reasonably prudent physicians” poses no particular challenge to assessing clinical judgments via an objective standard, as the “reasonably prudent physician” will (likely) view any of the treatment options that stand in clinical equipoise as being medically appropriate.

The type of disagreement that exists in cases of clinical equipoise is, however, fundamentally different than the type of disagreement one gets when disagreement results from distinct sets of physician values. Unlike in cases of clinical equipoise, physicians are likely to view other courses of action as both morally and medically impermissible. For example, the physician who refuses to provide the procedure in *Blood Transfusion* and *Necrotic Hand*, respectively, likely thinks that it is inappropriate for *anyone* to proceed with the relevant surgeries. From this physician’s perspective, the vocation of medicine forbids physicians from being the likely cause of a patient’s death. By contrast, the physician who takes the opposite stance in *Blood Transfusion* is likely to think that refusing to provide the surgery is an inexcusable refusal to attempt to save the patient’s life. Similarly, the physician who is willing to move forward with the risky surgery in *Necrotic Hand* may well view refusal to perform the procedure as a form of impermissible parentalism that inappropriately prioritizes preventing harm over patient values. Furthermore, rooted in deeply held values, these disagreements may be effectively intractable.

Disagreement in cases of clinical equipoise may not pose a challenge to assessing clinical judgments via an objective standard. The same cannot, however, be said about cases of disagreement rooted in the values that physicians bring to the clinical encounter. With regard to the latter, but not the former, there is likely to be substantial and intractable disagreement amongst “reasonably prudent physicians” about what is morally and medically permissible. This is particularly the case for clinical judgments that, like those involving abortion, find themselves at the center of a maelstrom of ethical dispute.

## Implications for Texas’ Abortion Law

Consider a third scenario:

### Gestational Diabetes

A 30-year-old woman with an unwanted pregnancy develops gestational diabetes. Though she intended to carry the pregnancy to term, she now requests an abortion.

Though recent literature documents growing concerns regarding outcomes related to gestational diabetes, both maternal and fetal outcomes are generally good.^48^ *Gestational Diabetes* should thus be a straightforward case for Texas’ abortion law. Consequently, the existence of even minimal ambiguity regarding the legal permissibility of an abortion would constitute a significant problem for Texas’ abortion regulations.

Texas regulations allow for an abortion in those cases where, in the exercise of reasonable medical judgment, an abortion is necessary to prevent (i) a “serious risk of substantial impairment of a major bodily function,” or (ii) “a life-threatening physical condition.”^49^ An analysis of Texas’ abortion regulation can thus be divided into two distinct questions. First, can judgments regarding the existence of a “serious risk of substantial impairment of a major bodily function” be evaluated against an objective standard? Second, can judgments regarding the existence of “a life-threatening physical condition” be evaluated against an objective standard?

#### Against an Objective Standard of a “Serious Risk of Substantial Impairment”

The Texas Health and Safety Code leaves both “serious risk” and “substantial impairment” undefined. Consider each in turn. Can judgments of “serious risk” be assessed by reference to an objective standard of medical judgment?

Recent evidence indicates that gestational diabetes leads to a 40% increased risk for cardiovascular disease (CVD).^50^ On one hand, a 40% increased risk of CVD is substantial. On the other hand, only 20% of the control group experienced CVD, meaning that gestational diabetes predicts an 8% increase in absolute risk of CVD.^51^

Consider a pregnant patient who is the sole caregiver for three children, one of whom has level three autism and will never live independently. Death or serious impairment means her children will likely be entrusted to a foster system rife with abuse and neglect.^52^ For this patient, an additional 8% risk of CVD plausibly constitutes a “serious risk” of substantial impairment — far more is on the line than just the patient’s well-being. It would thus be appropriate for a physician to judge that gestational diabetes does constitute a “serious risk.”

Nonetheless, for other physicians, this judgment would be inappropriate. The judgment that an abortion is the only way to prevent a “serious risk of substantial impairment” is likely to result in the termination of the pregnancy. For a physician who accepts fetal personhood and thus believes that abortion constitutes a harm to a fetal person, it would be appropriate to judge that an 8% increase in absolute risk of CVD does not constitute a “serious risk.”

In a case like *Gestational Diabetes*, there is no answer to the question: according to “reasonable medical judgment,” is an abortion necessary to prevent serious risk to the patient? As a putatively objective standard, reasonable medical judgment is not supposed to depend on the state of mind of the physician. Yet on the contemporary understanding of clinical decision-making, medical judgments about what constitutes a “serious risk” are responsive to physician values.

The same holds regarding judgments about “serious impairment.” Gestational diabetes is associated with increased risk of retinopathy.^53^ For many patients, the degree of retinopathy likely to result from well-controlled gestational diabetes does not constitute a “substantial impairment.” But consider a Navy pilot for whom even comparatively minor loss of vision could force a change of career.^54^ The shift from military to civilian life may entail not just the loss of a job, but the simultaneous loss of housing, childcare, and social safety net. Given the substantial losses associated with retinopathy, for many physicians, it would be appropriate to judge that even well-managed gestational diabetes poses a serious risk of substantial impairment for this patient. However, for a physician deeply committed to bringing about an abortion only when one is absolutely necessary, it would be appropriate to judge that, even for this patient, gestational diabetes does not present a serious risk of “substantial impairment.”

Clinical determinations regarding whether a pregnancy “poses a serious risk of substantial impairment of a major bodily function” are often, and appropriately, dependent on a physician’s views about the nature of harm and benefit. In such cases, medical judgments cannot be assessed against an objective standard of “reasonable medical judgment.” Thus, even regarding situations that should be straightforward, like *Gestational Diabetes*, Texas abortion regulations are profoundly ambiguous.

#### Against an Objective Standard of a “Life-Threatening Condition”

Texas abortion regulations also allow for abortions that are necessary to save a patient from a “life-threatening condition.” The Texas Health and Safety Code defines “life-threatening” as “a condition from which the likelihood of death is probable unless the course of the condition is interrupted.”^55^ Gestational diabetes is not often “life-threatening.” Here, an objective standard of medical judgment is on comparatively solid ground.

Yet physician values remain relevant, even to judgments about probability of death. In his seminal article on the role of values in science, Richard Rudner writes:

[S]ince no scientific hypothesis is ever completely verified, in accepting a hypothesis the scientist must make the decision that the evidence is sufficiently strong or that the probability is sufficiently high to warrant the acceptance of the hypothesis.… [I]f the hypothesis under consideration were to the effect that a toxic ingredient of a drug was not present in lethal quantity, we would require a relatively high degree of confirmation or confidence before accepting the hypothesis—for the consequences of making a mistake here are exceedingly grave by our moral standards.^56^

Consider two physicians, one who believes in fetal personhood and another who accepts the Jewish theological view that life begins at birth.^57^ Even given identical evidence regarding the probability of death from a gestational complication, these physicians may disagree about whether the condition is “life-threatening.” For the physician who believes in fetal personhood, the evidence may not be strong enough to justify the judgment that the condition is life-threatening; for the physician who believes that life starts at birth, the evidence may be plenty strong.

Importantly, this is not to suggest that the risks posed by a pregnancy complication are dependent on, e.g., one’s views about the permissibility of abortion. The risks posed by a pregnancy complication are independent of what anyone thinks or feels about them. Nonetheless, because their values play an ineliminable role in the reasonably prudent physician’s assessment regarding whether available evidence is strong enough to justify the conclusion that a pregnancy complication is life-threatening, many such judgments cannot be assessed via an objective standard.

This is not to deny that, in many cases, an objective standard can be used to assess judgments regarding whether a pregnancy complication is life-threatening. It will sometimes be the case that available scientific evidence is so overwhelming that the influence of values on epistemic judgments is trumped, and all reasonably prudent physicians will agree that a pregnancy is, or is not, life-threatening. But in many other cases, there will be an evidential penumbra around the probability that a condition is life-threatening. Within this penumbral space, the evidence will not be so overwhelming that it compels agreement amongst reasonably prudent physicians. In these cases, physician values will play a key role in shaping medical judgment regarding whether a patient has a “life-threatening condition.” In such cases, Texas’ current abortion regulations are profoundly ambiguous.

## A Potential Work-Around

It may seem that there is an easy solution to this challenge. Medical judgments involve at least three components: an assessment of the available evidence, responsiveness to patient values, and finally responsiveness to physician values. Of these, the first two can be assessed via an objective standard. Only the third and final component of medical judgment requires assessment via a subjective standard. This fact suggests a potential solution to the challenge physician values pose to assessing medical judgments via an objective standard: legislatures or courts could decide to define “medical judgment” solely in terms of the first two components, each of which can be assessed via an objective standard. If “medical judgment” is defined in this way, then, by definitional fiat, physician values become irrelevant to the legal assessment of “reasonable medical judgment,” and the concomitant challenge to assessing medical judgments via an objective standard is resolved.^58^

Even if such a redefinition of “medical judgment” were to be found constitutional, it would have profound implications for the quality of care that could be provided. One need not accept shared decision-making as the correct model of the physician-patient relationship in order to accept the view that physician values should inform medical judgment. However, in virtue of shared decision-making holding as a goal “build[ing] a consensus”^59^ or coming to “a shared understanding”^60^ between physician and patient, the shared decision-making model entails the relevance of physician values for medical judgment. Furthermore, shared decision-making is understood to be the “the pinnacle of patient-centered care.”^61^ Thus, redefining “medical judgment” in a way that is incompatible with the practice of shared decision-making would be to deny patients access to “the pinnacle of patient-centered care.”^62^

It is, furthermore, unlikely that such a redefinition of “medical judgment” would pass constitutional muster. Due process requires “that the law cannot compel the impossible.”^63^ But as Drescher argues, it is likely impossible to practice medicine in a way divorced from the values of the physician.^64^ Judgments about harm and benefit are fundamentally value judgments. Furthermore, judgments about what harms or benefits a patient are central to the practice of medicine; there is thus no practice of medicine without value judgments on the part of the physician. Redefining “medical judgment” so as to exclude physician values would thus not only be at odds with best practice in the form of shared decision-making, it would likely further demand the impossible from physicians and, as such, fail muster under the due process clause.

## Subjective Judgments in Abortion Law

Texas law bans abortions except when, as assessed by an objective standard of “reasonable medical judgment,” a pregnancy is life-threatening or presents a serious risk of substantial impairment. Yet on the understanding of clinical judgment foundational to contemporary medicine, physician values are central to medical judgment, and medical judgments can thus not be assessed via an objective standard. Texas law is consequently ambiguous regarding when deteriorating patient health renders abortion legally permissible.

Unlike Texas’ abortion regulations, Ohio assesses physician judgment via a subjective standard, i.e., “the professional judgment of the pregnant patient’s treating physician.”^65^ Ohio law thus leaves room for the central role of physician values in clinical judgment. As such, Ohio’s law, and laws relying on subjective standards for assessing physician judgments more generally, avoid the challenges explored herein.

It nonetheless remains unclear whether Texas abortion regulations, and similar regulations that rely on an objective standard of medical judgment, can be rescued by shifting to a subjective standard of medical judgment. Recent work has highlighted the extent to which medical judgments are nearly always made under conditions of uncertainty. As such, physicians will rarely judge that a condition is, e.g., “life-threatening.” Rather, medical decisions are informed by judgments regarding what diagnoses and prognoses are likely, given available evidence. If these analyses are correct, then, regardless of whether one relies on an objective standard or subjective standard of medical judgment, one will not find that physicians judge that pregnancy complications are life-threatening (only “likely life-threatening” or “potentially life-threatening”).^66^

Assessing these critiques falls outside of the scope of the manuscript. We have argued that the use of an objective standard of medical judgment can, and does, introduce extensive ambiguity into abortion regulation. Whether reliance on subjective standards of medical judgment introduces similar ambiguity remains an open question, though the contemporary literature indicates that a shift to subjective standards will not resolve the problem.

## Conclusion

In a recent editorial, one of the authors of the Texas Heartbeat Bill argued that maternal deaths resulting from physician inaction in the face of life-threatening pregnancy complications is best explained by the fact that “[p]ro-abortion groups and their allies in the left-wing media have been relentless in their attempts to scare doctors, pregnant mothers and the general public into believing that Texas law prevents doctors from helping patients and saving lives.”^67^ The analysis herein suggests a different diagnosis.

The problem arises from a gap between physicians’ understanding of medicine and the understanding of medicine that appears to be dominant amongst legislators and the courts. Texas abortion regulations appear to assume that appropriate clinical decision-making “is [solely] determined by value-neutral conventional, scientific, or technical characteristics.”^68^ Yet this is a view of medical judgment long ago rejected by medicine as impermissibly parentalistic.

Insofar as a misunderstanding of medicine lies at the heart of the problem, advocacy and educational outreach are likely the only solution. Individual physicians and state-level healthcare organizations should take steps toward educating representatives and judges about the contemporary understanding of clinical decision-making and the foundational principles that underlie modern medical practice. National organizations should support state-level outreach by designing and disseminating educational materials that physicians can use to guide these conversations. Short of such efforts, physicians may increasingly find themselves beholden to legal constructs built upon a misunderstanding of contemporary medical practice.
